# Scapular dyskinesis after breast reconstruction surgery for breast cancer: a retrospective clinical analysis on 67 patients

**DOI:** 10.1007/s12306-025-00904-x

**Published:** 2025-05-08

**Authors:** L. Murena, F. Santovito, A. de Grazia, G. Libretti, G. Galeazzi, G. B. Sidoti, N. Renzi, B. Trobec, A. Buoite Stella, V. Ramella, G. Papa, G. Canton

**Affiliations:** 1 Orthopedics and Traumatology Unit, Department of Medicine, Surgery and Health Sciences, University of Trieste, Strada di Fiume 447, 34149 Trieste, Italy; 2 Department of Medicine, Surgery and Health Sciences, University of Trieste, Strada di Fiume 447, 34149 Trieste, Italy; 3 Single-Cycle Master’s Degree Course in Medicine and Surgery, Department of Medicine, Surgery and Health Sciences, University of Trieste, Strada di Fiume 447, 34149 Trieste, Italy; 4 Plastic and Reconstructive Surgery Unit, Department of Medicine, Surgery and Health Sciences, University of Trieste, Strada di Fiume 447, 34149 Trieste, Italy

**Keywords:** Scapular dyskinesis, Scapula, Breast reconstruction, Rehabilitation, Shoulder

## Abstract

**Purpose:**

Mastectomy and breast reconstruction surgery are often associated with postoperative pain and functional limitation at the ipsilateral shoulder, potentially leading to scapular dyskinesis. However, few studies have determined how the type of surgery and rehabilitation might affect the development of such clinical condition.

**Methods:**

A retrospective observational study was performed on a clinical database of females who underwent surgical and adjuvant disease control treatment against breast cancer. Data included in this analysis were: demographics and clinical history, type of surgery and duration of physiotherapy, complications, as well as scapulohumeral rhythm and shoulder soreness evaluated during the orthopedic visit.

**Results:**

Based on the inclusion and exclusion criteria, 67 females (age 52 y, range 30–69) entered the statistical analysis. Static dyskinesis was present in 64.2% of the sample at the time of the visit, and it was found present bilaterally in 29.9% of the sample, whereas dynamic dyskinesis was found in 73.1% of the sample at the time of the visit. Longer physiotherapy (> 20 sessions) showed a trend for a lower risk of dynamic dyskinesis (OR 0.228, 95% CI 0.046–1.114, *p* = 0.072), and compared to the Subpectoral Tissue Expander, Prepectoral Implant-Based Breast Reconstruction presented a reduced risk for dynamic dyskinesis (OR 0.265, 95% CI: 0.074–0.952, *p* = 0.042).

**Conclusion:**

These preliminary findings suggest that some factors, such as the type of surgery and physiotherapy, might influence the development of scapular dyskinesis in females who undergo mastectomy and breast reconstruction.

## Introduction

Upper limb function requires adequate mobility of the shoulder, including the scapula, and efficient neuromuscular coordination (Michener et al. 2003). Scapular dyskinesis is an alteration in the normal position or motion of the scapula during coupled scapulohumeral movements [1] and is often associated with shoulder pain and injury [2–4]. Literature varies on whether it represents a cause or symptom of shoulder pathology, but it’s believed to be a risk factor for further injury [5].

Scapular dyskinesis is highly prevalent in the asymptomatic general population [6]. Alterations in scapular position and movement occur in 68% to 100% of patients with shoulder injuries [7]. It can be a consequence of several factors. The most common include neurological damage, thoracic kyphosis, degenerative muscle changes, instability and trauma outcomes of the acromioclavicular joint [1]. Other iatrogenic causes include non-orthopedic surgeries around the shoulder, especially mastectomy. Adhesive capsulitis is a relevant potential confounder in patients with shoulder dysfunction post-breast surgery. This condition is characterized by pain and significant restriction in the shoulder’s range of motion, which can complicate the assessment of scapular dyskinesis and other functional impairments. Given its frequent association with post-surgical immobility and altered biomechanics, adhesive capsulitis may impact scapular movement patterns and contribute to overlapping symptoms, thus influencing the overall interpretation of shoulder dysfunction [8]. Nowadays, autologous and prosthetic reconstruction are the most applied techniques in breast reconstruction following nipple-sparing or skin-sparing mastectomy [9–11]. In particular, the use of implants remains the most common method [9]. Prosthetic reconstruction can be performed in a variety of ways that include one-stage reconstruction with a direct implant and two-stage reconstruction with a tissue expander followed by its substitution with a definitive implant. In addition, implants can be placed under the pectoralis muscle (subpectoral), partially under the pectoralis muscle (partial subpectoral), or above it, with the use of acellular dermal matrix (ADM) to protect the implants (prepectoral) [12]. The choice of the best reconstructive method is tailored to every patient, considering the thickness of mastectomy flaps, possible risk factors such as smoking habits, diabetes, BMI, previous radiotherapy, and the eventual need for postoperative radiotherapy [9, 12, 13].

The widely recognized negative consequences of mastectomy and breast reconstruction surgery include pain, limited mobility, and altered body image. On the other hand, the potential influence of these procedures on scapular movement remains a less explored area. The loss of a breast produces soft tissue asymmetry and mass distribution through the chest wall, which may affect upper limb motion resulting in shoulder discomfort [14]. The limited number of literature studies reporting the relationship between scapular dyskinesia and mastectomy find high incidence values, ranging between 47.5 and 70% [15].

Scapular dyskinesis could have far-reaching consequences for these women, impacting not only their immediate postoperative experiences but also their long-term quality of life. While the association between mastectomy and scapular dyskinesis has been previously reported in the literature, there is no study to our knowledge that analyzes the presence of pathological alterations in scapulohumeral rhythm and shoulder soreness in breast reconstruction surgery, either delayed or immediate. In detail, it is unclear whether reconstruction can positively affect scapular kinetics concerning mastectomy or whether the occurrence of scapular dyskinesis may be also associated with reconstruction. Therefore, this study aimed to assess the prevalence of dyskinesis in patients undergoing breast reconstruction after mastectomy. A secondary aim of the study was to evaluate any association with surgery type, rehabilitation, and postoperative complications.

## Materials and methods

Sixty-seven patients were enrolled who underwent surgical and adjuvant disease control treatment against breast cancer (Cattinara Hospital in Trieste, ASUGI) from 2019 to 2022. Patients older than 72 years, with severe disabilities, and who underwent quadrantectomy with breast conservation were excluded. The recruited patients were still in a postoperative follow-up status. All the procedures performed in this study were conducted in accordance with the ethical standards of the institutional and/or national research committee and with the 1964 Helsinki Declaration and its later amendments or comparable ethical standards. The study was approved by the Bioethics Committee of the Medical University of Trieste. An informed consent was obtained from all individual participants included in the study and they have consented to the submission of the study to the journal.

The first part of the examination consisted of collecting the medical history regarding the disease and its treatment, recording the type of mastectomy and reconstruction performed, the lymph node surgery performed, the surgical timing, any postoperative complications, postoperative pain, postoperative physiotherapy, and adjuvant treatment (hormonal, chemotherapy, radiotherapy). A remote personal pathological history of each patient regarding the presence of autoimmune diseases, rheumatological disorders, diabetes, and fibromyalgia was recorded. The second part of the visit involved a specialized orthopedic assessment including a second evaluation on the presence of cervicalgia and low back pain, positive history of upper extremity trauma and possible treatment, pain at the time of the visit and possible localization, and physiotherapy treatment for the conditions listed above if any. Objective examination followed, with inspection aimed at detecting a pattern of scapular dyskinesis in a static position. If present, classification of the detected dyskinesis was performed according to previous literature [16] (Table 1). Then, an evaluation of the active range of motion (RoM) during elevation and abduction was performed. Other shoulder movements were assessed in the different planes [4, 17]. The presence of a pattern of dynamic scapular dyskinesis was simultaneously assessed. Detected active dyskinesis was classified according to Kibler et al. indicating the affected side [1]. Scapular dyskinesia in our research was assessed by specifically trained physicians, using the parameters established by Kibler and McMullen (2003) [1]. Palpation, researching tender points, was performed on the coracoid and biceps brachii. If present, the symptomatologic pattern suggesting a SICK Scapula Syndrome, characterized by the presence of scapular malposition, prominence of the medial inferior angle of the scapula, pain on palpation of the coracoid process and documented scapular dyskinesis [18] was recognized and registered.

**Table 1:** Scapular dyskinesis classification

Type I	Prominent inferior medial scapular border
Type II	Prominent entire medial scapular border
Type III	Excessive superior migration of superior medial scapular border
Type IV	Normal and symmetric scapular motion

The overall prevalence of scapular dyskinesis at rest and during movement and according to the type of mastectomy and breast reconstruction performed, number of postoperative physiotherapy sessions performed, adjuvant therapies (hormone therapy, chemotherapy, and radiotherapy) prevalence of cervicalgia, surgical time and intraoperative limb positioning, and prevalence of Web Axillary Syndrome as a postoperative complication were then reported. More specifically, the physiotherapy protocol was standardized according to the internal consensus between the orthopedic surgeons and the physiotherapists and based on previous works [4], consisting in individual sessions lasting 45–60 min each, at least 2 times per week, combining manual therapy techniques and active exercises. The protocol focused on restoring the RoM of the shoulder, which is often reduced because of surgery or immobilization, therefore specific exercises, including proprioceptive neuromuscular facilitation (PNF) techniques such as hold–relax and contract–relax (post-isometric relaxation), were applied to all movement directions of the shoulder (anterior flexion, abduction, external rotation, and internal rotation) to restore function, strength and motion control; the second part was targeted to regain pectoralis minor flexibility, often tightened or restricted, affecting both posture and shoulder mechanics, including proprioceptive exercise on the scapula to increase awareness of the scapular movements in the medial and lateral spaces, above and below, as well as circular, straight, and curved trajectories were used first without weights and progressively with weights and rubber bands. Physical therapy was also necessary to reduce pain and give patient comfort after surgery, but also manual lymphatic drainage was incorporated in the program to reduce swelling and to support lymphatic system both linked to surgery and immobilization.

### Statistics and data analyses

All statistical analyses were performed with SPSS version 23 (IBM). This is the primary analysis of these data. Data are reported as the means, standard deviation, counts and proportions (%) as appropriate. To provide a preliminary statistical analysis of the association between clinical, surgical and dyskinesis characteristics, nonparametric correlations were performed using Spearman’s rank correlation coefficient (*ρ*), and binary logistic regressions were performed considering static and dynamic dyskinesis as the dependent variable. In addition, individuals with and without static/dynamic dyskinesis were compared with the Mann–Whitney U-Test and Fisher’s exact test. Significance was set for *p* < 0.05.

## Results

Data from sixty-seven females (age 52 y, range 30–69) were retrieved according to the inclusion and exclusion criteria, and therefore entered the final analysis.

### Clinical and surgical outcomes

Nipple-sparing mastectomy (NSM) and skin-sparing mastectomy (SSM) were the most prevalent techniques (44.8% and 52.2% of the sample, respectively), and were almost equally performed on the left (35.8%), right (34.3%) and bilateral (29.9%) breast. Considering the type of surgical reconstruction, the prevalent technique was the Subpectoral Tissue Expander (64.2%). Additional clinical and surgical characteristics are shown in Table 2. Despite only 32.8% of the sample reported severe pain after surgery, 22.4% also reported pain during active movement at the follow-up visit, as well as neck pain (16.4%). Postoperative physiotherapy was performed by all the included patients, with only 10.4% of the sample requiring more than 20 sessions.

**Table 2:** Clinical and surgical characteristics of the sample. Data expressed as mean ± standard deviation, counts and proportions (%)

	Static			Dynamic		
	No (*n* = 24)	Yes (*n* = 43)	Sig	No (*n* = 18)	Yes (*n* = 49)	Sig
Age [years]	55.0 ± 7.7	50.7 ± 9.3	0.058	52.7 ± 10.9	52.1 ± 8.3	0.828
*Type of mastectomy* [*n* (%)]			0.142			0.573
NSM	8 (33.3)	23 (54.5)		10 (55.6)	21 (42.9)	
SSM	15 (63.0)	20 (46.5)		8 (44.4)	27 (55.1)	
SRM	1 (4.7)	0 (0.0)		0 (0.0)	1 (2.0)	
*Type of reconstruction* [*n* (%)]			0.783			0.113
ExpSubpectoral	14 (58.3)	29 (67.4)		9 (50.0)	34 (69.4)	
ExpPrepectoral	4 (16.7)	4 (9.3)		1 (5.6)	7 (14.3)	
ProtSubpectoral	1 (4.2)	1 (2.3)		1 (5.6)	1 (2.0)	
ProtPrepectoral	5 (20.8)	9 (21.0)		7 (38.8)	7 (14.3)	
Web axillary syndrome [*n* (%)]	3 (12.5)	10 (23.3)	0.286	2 (11.1)	11 (22.4)	0.298
> 20 physiotherapy sessions [*n* (%)]	4 (16.6)	3 (7.0)	0.214	4 (22.2)	3 (6.1)	0.056

NSM, nipple-sparing mastectomy; SSM, skin-sparing mastectomy; SRM, skin-reducing mastectomy

Statistical significance reported at the Mann–Whitney U-Test and Fisher’s Exact test, respectively

### Dyskinesis prevalence and characteristics

**Fig. 1:**
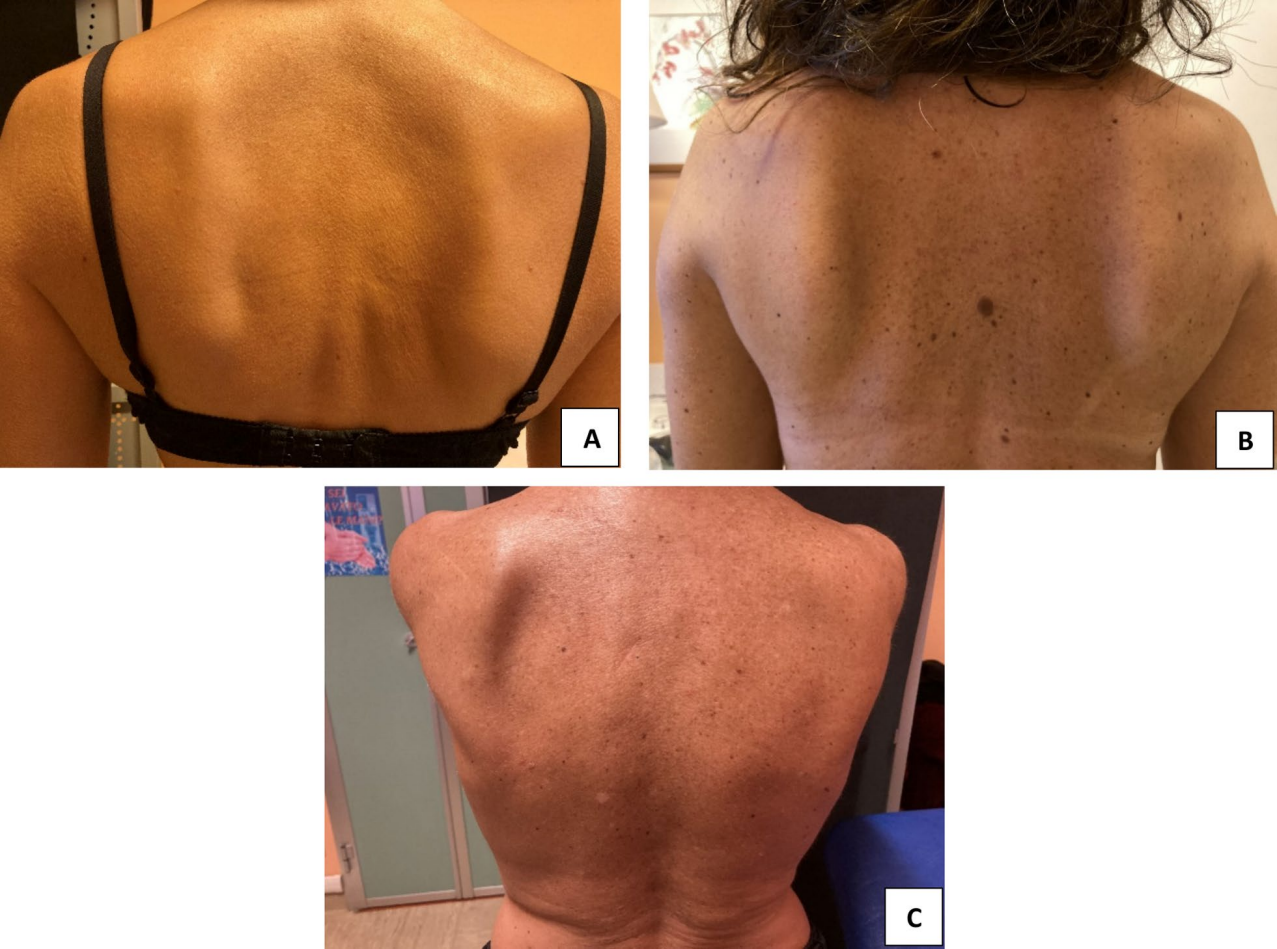
**A** Prominent inferior medial scapular border, classified as type I dyskinesis. **B** Prominent entire medial scapular border, classified as type II dyskinesis. **C** Excessive superior migration of superior medial scapular border, classified as type III dyskinesis

Static dyskinesis was present in 64.2% of the sample at the time of the visit, and it was found present bilaterally in 29.9% of the sample; interestingly, on the right side type 1 dyskinesis was more common (28.4% of the total sample), whereas type 2 dyskinesis was more common on the left side (29.9% of the total sample). Pain during coracoid and biceps brachii palpation was commonly absent (71.6% and 91.0% respectively). Dynamic dyskinesis was found in 73.1% of the sample at the time of the visit, and similarly to static dyskinesis, type 1 was more common on the right side (16.4%) and type 2 on the left side (31.3%). We found no significant different proportion of patients with different type of mastectomy in those with and without dyskinesis (Table 2). SICK scapula syndrome was found in only 6% of the total sample, and web axillary syndrome was found in 19.4% of the sample. Additional dyskinesis-related outcomes are shown in Table 3 and examples of scapular dyskinesia are shown in Fig. 1.

**Table 3:** Scapular dyskinesis characteristics of the sample. Data expressed as mean (range), counts and proportions (%)

*n* = 67	
*Static scapular dyskinesis*, *n* (%)	43 (64.2)
Type I	19 (28.4)
Type II	20 (29.9)
Type III	1 (1.5)
*Dinamic scapular dyskinesis*, *n* (%)	49 (73.1)
Type I	11 (16.4)
Type II	21 (31.3)
Type III	12 (17.9)

### Associations between clinical/surgical characteristics and dyskinesis

Static dyskinesis was found to be significantly correlated with age (Spearman’s rho = − 0.283, *p* = 0.020). In contrast, no significant correlations were found between dynamic dyskinesis and the collected outcomes, although a tendency was found with the length of physiotherapy treatment (Spearman’s rho = − 0.233, *p* = 0.057). Indeed, when the length of physiotherapy (higher or lower than 20 sessions) was entered in the logistic regression, longer physiotherapy showed a trend for a lower risk of dynamic dyskinesis (OR 0.228, 95% CI 0.046–1.114, *p* = 0.072). Finally, when the type of reconstruction was analyzed, compared to the Subpectoral Tissue Expander, Prepectoral Implant-Based Breast Reconstruction suggested a reduced risk for dynamic dyskinesis (OR 0.265, 95% CI 0.074–0.952, *p* = 0.042) (Table 4).

**Table 4:** Associations between clinical/surgical characteristics and scapular dyskinesis

Association between clinical, surgical and dyskinesis characteristics	Significance
Rehabilitation (higher than 20 physiotherapy sessions)	Spearman’s rho = − 0.233, *p* = 0.057
Longer physiotherapy/ lower risk of dynamic dyskinesis	OR 0.228, 95% CI 0.046–1.114, *p* = 0.072
Type of reconstruction (Prepectoral implant-based breast reconstruction)	OR 0.265, 95% CI 0.074–0.952, *p* = 0.042

## Discussion

In the literature, the prevalence of scapular dyskinesis in patients who underwent mastectomy was reported between 47.5 and 70% [15]. To our knowledge, however, there is no previous literature report about the association of the prevalence of dyskinesis in mastectomized patients who underwent breast reconstruction. In the present study the prevalence of scapula dyskinesis in patients undergoing breast reconstruction after mastectomy proved to be high. In detail, a prevalence between 64.2% (Static evaluation) and 73.1% (Dynamic evaluation) was found, with type 1 dyskinesis being the most common. Even though there is a lack of studies in the literature evaluating scapular dyskinesis following breast reconstruction, the present study found a comparable incidence of dyskinesis. Possible explanations proposed in the different studies are: shortness, tension, or stiffness of the pectoralis minor muscle, pain, inhibition of muscle activation, and edema after surgery; complications, such as lymphedema resulting in a feeling of heaviness in the arm and upper limb limitations cause increased pain sensitivity in the trapezius and deltoid muscles.

The reported explanations might all be applied to the present study population. In detail, involvement of the serratus anterior muscle during breast reconstruction surgery might be expected, both due to the imbibition of the tissues and the possible development of a fibrotic component, possibly leading to scapular dyskinesis. To a lesser extent, the pectoralis minor may also be involved. These considerations might better apply to two-stage reconstruction techniques. When the choice is to proceed with a two stages reconstruction, a tissue expander previously inflated with saline solution is positioned in the pocket created under the pectoralis muscle and the serratus anterior laterally, after the incision of the fascia of the pectoralis muscle along its lateral border. Then the pocket is sutured with the tissue expander inside it. In the postoperative period, about every three weeks, the expander is gradually inflated with saline solution to reach the desired volume and then substituted with the definitive implant.

Possible early complications of this type of reconstruction are hematoma, infection, and seroma, while the most common late complications are pain and discomfort, animation deformity, capsular contracture, leak or rupture. Conversely, in one-stage reconstruction, the implant is placed in the prepectoral pocket after covering the surface of the implant with ADM to help its integration. In this setting, the muscles are not employed to create the pocket that hosts the implant, and this is associated with lower comorbidities. Frequent complications are seroma, infection, hematoma, and loss of the implant especially in case of skin necrosis [19, 20]. In addition, the effect of the partial immobilization of the ipsilateral upper limb to which patients are subjected after breast reconstruction is not negligible, both as a reaction to pain and to avoid possible surgical wound complications.

In the present study, Prepectoral Implant-Based Breast Reconstruction compared to the Subpectoral Tissue Expander suggested a reduced risk for dynamic scapular dyskinesis (OR 0.265, 95% CI 0.074–0.952, *p* = 0.042). Some reasons why a prepectoral implant might be preferred over a subpectoral expander are greater softness and naturalness because the prosthetic tissue is placed over the pectoral muscle and closer to the skin; faster postoperative recovery, because prepectoral implant reconstruction does not require the creation of space under the pectoral muscle as is done with subpectoral expanders, postoperative recovery may be faster and less painful; lower risk of muscle complications, such as capsular contractures or muscle disorders is reduced and simpler surgical procedure, where the position above the pectoralis muscle makes the prepectoral implant more easily accessible during surgery. However, it is important to keep in mind that there are also situations in which reconstruction with a subpectoral expander may be preferable, such as for some patients with little skin coverage or for whom a more gradual tissue expansion needs to be used. Ultimately, the choice between reconstruction with a prepectoral implant and reconstruction with a subpectoral expander will depend on the patient’s specific circumstances and preferences, and it is best to discuss this in detail with the plastic surgeon to evaluate the available options.

A relevant role in preventing scapular dyskinesis development may be played by rehabilitation. Literature suggests a positive effect of rehabilitation on scapular motion. Specific exercises for scapular rehabilitation include flexibility exercises to decrease scapular traction, and scapular stabilization exercises to optimize scapular kinematics [21]. Regarding stretching exercises, to reduce scapular traction, kinesitherapy with shoulder horizontal abduction at 90° and 150° of elevation has been demonstrated to be useful in increasing pectoralis minor flexibility and the external rotation and posterior tilt of the scapula during forward elevation [21–23]. As regards scapular stabilization exercises, it is important to consider the role of the serratus anterior and trapezius muscles that act as scapular stabilizers. The serratus anterior plays an essential role in determining scapular ER and posterior tilt, and the lower trapezius contributes to stabilizing the scapular position. Scapular stabilization exercises are based on closed and open kinetic chain exercises, including push-ups on a stable or unstable surface, lawnmower exercises and resisted scapular retraction [21, 24, 25]. The consensus suggests potential advantages of integrating structured exercise programs into recovery protocols. In addition, physical rehabilitation showed significant improvement in shoulder movement and strength improvement with gradual intensity exercises, and early exercise was more effective than delayed exercise. More importantly, exercise did not increase the risk or change the incidence rate of lymphedema. Early exercise (compared with delayed exercise) also helps wound healing by increasing the volume of wound drainage. These results indicate that exercise could play a crucial role in improving both physical and emotional well-being [26, 27]. In the present study, longer rehabilitation after surgery (more than 20 physiotherapy sessions) showed a trend for a lower risk of dynamic dyskinesis (OR 0.228, 95% CI 0.046–1.114, *p* = 0.072). The association was not statistically significant, but there was a clinically observable difference between the two groups of patients, and it can be hypothesized that a higher number of patients would help to identify a statistical significance; as such, despite preliminary and cautiously interpreted, these findings are encouraging also based on the possible clinical translational approach. Indeed, it is possible to identify an inverse correlation between the duration of physiotherapy and the incidence of scapular dyskinesia following breast reconstruction surgery. These results underline the importance of physical therapy after breast reconstruction and mastectomy, which might also aim to recover physiologic shoulder and scapular motion. Considering the average age of the patients undergoing breast reconstruction surgery, a good goal could be rehabilitation, where there is concurrent activation of muscles to perform activities of daily life [28].

**Fig. 2:**
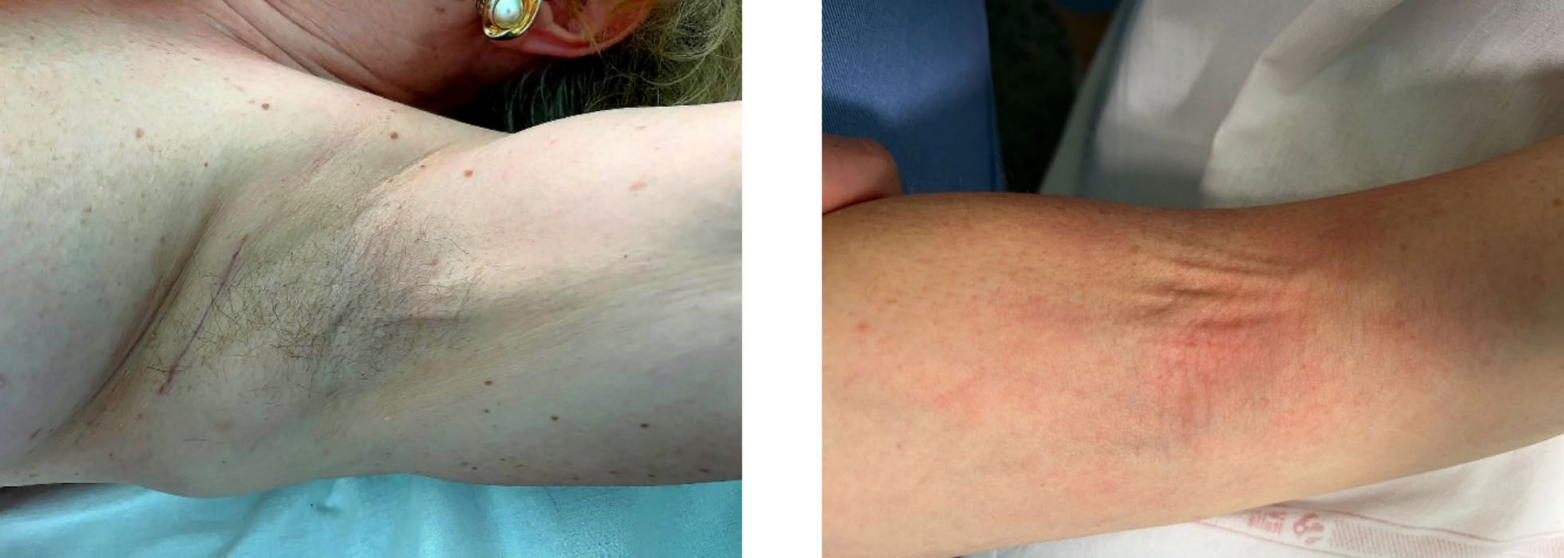
The axillary web syndrome under the axilla and in antecubital fossa

As far as postoperative complications are concerned, an association between dyskinesis and axillary web syndrome (AWS) was revealed, even if not significant. The AWS or lymphatic cording refers to a ropelike structure that develops mainly under the axilla that arises after axillary dissection and can extend to involve the medial aspect of the ipsilateral arm down to the antecubital fossa **(**Fig. 2**).**

The incidence after mastectomy and breast reconstruction is experienced by 6–86% of patients who have undergone axillary dissection surgery, with ROM restriction accused by 74% of subjects [29–32]. The possible influence of this complication on scapular motion may reside in the retraction of the periscapular soft tissues, also associated with pain and skin imbibition, with consequent alteration of both active and passive mobility of the scapula, determining in some cases the onset of static and dynamic dyskinesis. Since there are currently no other reports and studies in the literature on possible associations between dyskinesis and axillary web syndrome, an effective comparison cannot be made at this time; however, we hope that these preliminary results could be compared with future research to better describe the prevalence and characteristics of this condition.

Among the limitations of the present study, the relatively small size of the sample being analyzed (which is consistent with other studies already present in the literature) and the retrospective design of the study suggest caution when interpreting these findings and their generalization. The absence of a control group indeed limits comparative insights and it represents one of the limits of the present study. However, our study is based on the protocols of our hospital, in which is standard practice to always follow mastectomy procedures with reconstruction to give the patients the best standard of care. Moreover, the inclusion and exclusion criteria have been adopted to limit the confounders and aimed to provide a novel overview of the possible factors affecting scapular function after breast reconstruction surgery for breast cancer.

Postoperative scapular dyskinesis proved to have a high prevalence after breast reconstruction, reaching values between 64.2 and 73.1%, with type 1 dyskinesis being the most common. Longer postoperative rehabilitation, as well as Prepectoral Implant-Based Breast Reconstruction compared to the Subpectoral Tissue Expander, seems to have a protective role on scapular motion alterations incidence, while the development of axillary web syndrome seems to have a negative effect. Further studies with a larger number of patients involved may reveal other associations and detect possible risk factors that may affect the incidence of scapular dyskinesis in these patients.

## Data Availability

Anonymized data can be requested upon reasonable request to the corresponding author.
